# Luria revisited: cognitive research in schizophrenia, past implications and future challenges

**DOI:** 10.1186/s13010-015-0026-9

**Published:** 2015-02-27

**Authors:** Yuliya Zaytseva, Raymond CK Chan, Ernst Pöppel, Andreas Heinz

**Affiliations:** National Institute of Mental Health/Prague Psychiatric Center, Topolova 748, 250 67 Klecany, Czech Republic; Human Science Centre and Institute of Medical Psychology, Ludwig-Maximilians-Universität, Goethestr. 31/1, 80336 Munich, Germany; Moscow Research Institute of Psychiatry, Poteshnaya str.3, 107076 Moscow, Russia; Institute of Psychology,Chinese Academy of Sciences, 16 Lincui Road, 100101, Chaoyang District, Beijing, China; Department of Psychology, Peking University, 5Yiheyuan Road, Beijing, 100871 China; Department of Psychiatry and Psychotherapy, Campus Charité Mitte, Charité-Universitätsmedizin Berlin, 10115 Berlin, Germany

**Keywords:** Luria, Neurocognitive test battery, Schizophrenia, Cognitive deficits, Taxonomy of functions, Brain connectivity, Temporal perception

## Abstract

Contemporary psychiatry is becoming more biologically oriented in the attempt to elicit a biological rationale of mental diseases. Although mental disorders comprise mostly functional abnormalities, there is a substantial overlap between neurology and psychiatry in addressing cognitive disturbances. In schizophrenia, the presence of cognitive impairment prior to the onset of psychosis and early after its manifestation suggests that some neurocognitive abnormalities precede the onset of psychosis and may represent a trait marker. These cognitive alterations may arise from functional disconnectivity, as no significant brain damage has been found. In this review we aim to revise A.R. Luria’s systematic approach used in the neuropsychological evaluation of cognitive functions, which was primarily applied in patients with neurological disorders and in the cognitive evaluation in schizophrenia and other related disorders. As proposed by Luria, cognitive processes, associated with higher cortical functions, may represent functional systems that are not localized in narrow, circumscribed areas of the brain, but occur among groups of concertedly working brain structures, each of which makes its own particular contribution to the organization of the functional system. Current developments in neuroscience provide evidence of functional connectivity in the brain. Therefore, Luria’s approach may serve as a frame of reference for the analysis and interpretation of cognitive functions in general and their abnormalities in schizophrenia in particular. Having said that, modern technology, as well as experimental evidence, may help us to understand the brain better and lead us towards creating a new classification of cognitive functions. In schizophrenia research, multidisciplinary approaches must be utilized to address specific cognitive alterations. The relationships among the components of cognitive functions derived from the functional connectivity of the brain may provide an insight into cognitive machinery.

## Introduction

Recent developments in the neurosciences are bringing together psychiatry and neurology, which were separated for nearly a century. The successful search for biological markers for schizophrenia, such as functional and structural abnormalities of the brain [1], cognitive deficits [2], and minor neurological signs [3,4], have shifted psychiatry to a more biologically-oriented branch of medical science. The importance of understanding structural and functional abnormalities derives from its potential impact on behavioral performance and particularly on cognitive functioning. Thus, cognitive deficits in schizophrenia go beyond the presence of positive and negative symptoms in the prodromal phase of the illness [5,6], becoming more severe during the acute phase and remain in remission. Neuropsychological studies of schizophrenia have a long history, and substantial disturbances in motor and perceptual processes, spatial functions, verbal and non-verbal memory, executive functioning, and difficulties concentrating or maintaining attention have been demonstrated [7-11]. It has been repeatedly shown that the degree of cognitive deficit plays an important role in determining the prognosis of recovery in these patients and has a great impact on social functioning [12,13]. Despite extensive research, cognitive deficits have not been found to sufficiently distinguish between schizophrenia and schizophrenia spectrum disorders [14], hence, they were not incorporated into the criteria of the current Diagnostic and Statistical Manual of Mental Disorders, Fifth Edition (DSM-5) [15]. Could cognitive alterations in schizophrenia be specified in the same way as they are specified in neurology? Indeed, the clinico-anatomical approach dominant in neurology does not seem directly applicable to psychiatry. This important issue must be addressed.

Schizophrenia has often been considered as a brain disease. Structural neuroimaging has revealed a reduction in the intracranial volume with a similar decrease in the volume of white matter in schizophrenia. Regions most consistently showing clusters of decreased grey matter detected by voxel-based morphometry (VBM) are the insula, anterior cingulate cortex, inferior and medial frontal gyrus, middle and inferior temporal gyri, amygdala, and thalamus [16,17]. The decreased density of grey matter does not, however, exceed 2-3%, as suggested by longitudinal studies and these neuroanatomical alterations may be associated with antipsychotic treatment [18,19]. To date, there is no clear understanding of what the nature of cognitive deficits is in schizophrenia. On the one hand, the whole brain volume tends to correlate with general intelligence, as well as with a range of specific cognitive functions, as shown in comparisons between normal controls and schizophrenia patients [20]. From the topological view, the prefrontal cortex is associated with executive function. The temporal lobe, hippocampus, and parahippocampal gyrus correlate with cognitive abilities, such as performance speed and accuracy, memory, executive function, verbal competence and the ability to form abstract categories. Nevertheless, cognitive dysfunction can’t be fully explained by grey-matter loss, and the pattern of cognitive deficits seems to be more diverse than the pattern of structural alterations.

Alternatively, cognitive deficits might emerge from the altered functional connectivity of the brain. The “disconnectivity theory of schizophrenia” implies an abnormal pattern of connections among distinct brain regions [21]. This disrupted connectivity may involve either exaggerated connections or weakened pathways and result in altered functional integration [22,23]. Patients have revealed widespread functional connectivity deficits in a large network of brain regions which primarily affect connectivity between the frontal cortex and posterior regions and occur irrespective of task context [23]. It has also been shown that deficits in attention and working memory are correlated with distinct alterations in functional coupling, particularly hyperactivity of the default-mode network, suggesting schizophrenia-related dysregulation of inhibitory brain circuits [24,25]. The question remains whether we are able to identify cognitive markers based on functional disconnectivity in schizophrenia.

Despite growing empirical evidence of cognitive disturbances and structural and functional brain alterations in schizophrenia, their underlying mechanisms still seems difficult to uncover. This issue is related to a larger problem within cognitive neuroscience – the lack of a valid taxonomy of cognitive functions [26]. A strong theoretical framework is needed to make solid inferences about cognitive processes from structural and functional brain findings. An attempt to stratify cognitive functions was made by the eminent Russian neuropsychologist A.R. Luria^a^, who categorized cognitive functions into three functional blocks, each of which makes its equal and unique contribution to the cognitive machinery. His concept was utilized for many years as a basis for analyzing cognitive distortions in neurological patients and proved to be informative and predictive in terms of outcomes.

In the present review, we aim to combine the systematic approach proposed by Luria with contemporary attempts to systematize current knowledge in cognitive research and underline some challenges in neuropsychology in order to improve diagnostics of schizophrenia and related disorders.

## Review

### The main principles of Luria’s approach

In his book “Principles of Neuropsychology (The Working Brain)”, Luria proposed a model of cerebral organization which assumed a specific distinction of functions operating as components of functional systems [27]. Luria conceived the brain as divided into three principal blocks. The first block regulates arousal and the state of vigilance, providing the brain with a stable basis for the organization of its various processes. The components of the first block are located in the upper and lower parts of the brains stem and particularly in the thalamus, which controls wakefulness. The second block processes the receipt, analysis, and storage of information. The specific sensory inputs are analysed and integrated into more complex sensations, which are later synthesized into even more complex perceptions. These perceptions contain information coming from various sensory modalities and enable the construction of scenes. The second functional block includes temporal processing, i.e. the recognition of simultaneity or the succession of events [28,29]. Simultaneous processing assumes the arrangement of incoming information into a holistic pattern or gestalt that can be integrated or “surveyed” in its entirety (as in visual recognition). Successive processing refers to encoding information into a discrete, serial order where the detection of one portion of the information depends on its temporal position relative to other material (acoustic recognition, speech processing, reading, writing, etc.). The third functional block of the brain comprises the frontal lobes and addresses the formation of intention, programming, regulation, and control of behaviour. It is responsible for the performance of complex tasks and also monitors ongoing actions, comparing the effects of the actions taken with the initial intentions, as hypothesized, for instance, in the reafference principle and its potential applications [30-32].

Luria claimed that any form of psychological activity is a system involving the simultaneous operation of the three functional units, and he stressed that patients’ inability to perform a certain task does not necessarily specify an area of brain dysfunction because each behavioral task requires the coordinated and integrated activity of a number of cortical and subcortical areas, all contributing differently to the execution of the task [33,34]. In other words, at a phenomenological level, the psychological functions are not restricted to the single location in the brain but rather are distributed as different modular components across the system. This explanation of the relatedness of cognitive functions to neuroanatomy has its roots in the double dissociation theory, originally proposed by Hans-Lukas Teuber [35]. This theory postulates that independent functions are associated with independent anatomical substrates. In Luria interpretation, a particular area of the brain usually represents a single “factor”, that subserves a several different systems and supports various psychological functions. The pattern of interacting factors responsible for a given behavior is called a functional system. Each area of the brain participates in numerous functional systems, as has been demonstrated in numerous studies using imaging technology, like functional magnetic resonance imaging (fMRI) [eg. 36-38].

The concept of a syndrome, as an extension of these principles, was pivotal in Luria’s concept, whereby a syndrome was identified as a constellation of factors and symptoms. Thus, Luria’s clinical method entailed the accumulation of converging evidence from many tasks to define a syndrome. The syndrome, then, emerged as a common cognitive process that was disrupted in seemingly disparate tasks. This clinical method, in turn, allowed Luria to define various areas of regional specialization in the brain and to map sets of functional systems that operate at various levels of generality within and between these brain areas. The research process, that Luria utilized, involved the definition of a variety of syndromes by identifying one or more cognitive processes that are common to tasks performed by subjects with brain damage.

Luria’s diagnostic tests consist of numerous specific procedures which are designed to isolate dysfunctions; as compared to the more general assessment characteristics of other neuropsychological tests [for instance see 39-41]. It includes an evaluation of all the domains necessary for a complete neuropsychological examination (motor functions, sensory skills (auditory, tactile, and visual), verbal skills (expressive speech, receptive speech, reading, and writing), spatial skills, arithmetic abilities, memory, and intellectual skills. Further development and interpretation of neuropsychological tests introduced by Luria was subsequently continued by his students and successors [42-50].

Luria’s concept was considered to be a blueprint for the development of neuropsychological tests and also for the specification of cognitive functions. Naglieri & Dass [51] based their theory of human intelligence (PASS theory: Planning, Attention, Simultaneous, and Successive) on Luria’s approach. Their composition of the functional system comprising planning, attention, and simultaneous/successive processing attempted to integrate cognitive functioning and became an alternative approach to intelligence which had traditionally included only verbal, nonverbal, and quantitative tests. The authors claimed that intelligence has some prerequisites originating in basic cognitive processing and precludes verbal achievement tests, such as vocabulary. Brain functions are considered to be the building blocks conceptualized within a cognitive-processing framework. The PASS theory was operationalized in the Cognitive Assessment System (CAS) [52], and its constructs are strongly related to the achievement. One of the purposes of the CAS is to anticipate levels of academic performance on the basis of levels of cognitive functioning. The results provided some support for Luria’s approach [26].

### Luria’s test battery

Neuropsychological tests are traditionally divided into two main groups, quantitative (psychometric) tests, which are focused on the achievement of results in a standard set time, and qualitative, process-oriented tests targeting performance and qualifications of errors (Luria’s approach). Orientation towards achievement implies error detection and quantifies the degree of the impairment. However, when the testing procedure focuses on the process, one can then conceive the subject’s strategies, the difficulties that the subject might experience while performing the task, and predict the kind and amount of help a subject might need to successfully complete the task. Besides, Luria employed a “single-case” methodology compared to the orientation towards group statistics in psychometric studies. Luria’s approach is centred on the patient (on the specifics of his/her mental processes and personality), whereas the psychometric approach focuses on the disease or the defect. In the latter case, a patient is evaluated within a statistical continuum.

An essential feature of Luria’s tests is their synthetic evaluation of complex forms of cognition, such as speech, writing, or problem solving. This enables a dynamic analysis of difficulties discovered in tests [53]. An individualized, qualitative approach allows symptoms to be combined into syndromes in which deficits and preserved functions are qualified. As the procedure takes place in an ongoing phenomenological and dynamic interaction between examiner and patient, the patient benefits from feedback, as it provides the possibility for change and improvement in performance.

Moreover, different levels of cognitive processes, like direct sensorimotor actions and reactions mediated by the level of complex operations, or speech, can be addressed. This approach also makes it possible to extract specific information on the patient’s present cognitive capacities, which might be useful for designing an individual rehabilitation program and making optimal use of the remaining intact functions [54,55].

Despite these advantages, Luria’s tests have also been widely criticized. One significant drawback has been the lack of direct evaluation of the tests and controlled scoring. Previously, scoring was determined by the clinician’s personal assessment based on his/her experience and knowledge rather than other normative data. Standardized neuropsychological test batteries have demonstrated their greatest reliability and validity in patients with focal, well-defined neurological diseases. In these patients, the test profiles typically highlight focal areas of strengths and weaknesses in the functions of the brain. However, the clinical utility of standard tests batteries and their reliance on scaled score differences is limited when evaluating patients with severe or diffuse neurobehavioral disorders [56].

The development of methods for the quantitative assessment of Luria’s test results is based on analyses of the test structure. This western influence provides the advantages of psychometric methods (objectivity, reliability, validity, standardization) without sacrificing the deep insights of neuropsychology as developed by Luria. It was Charles Golden and his associates who attempted to standardize and quantify Luria’s methods with the Luria-Nebraska test [54]. This was welcomed by many, and translations were made into several languages. Luria’s ideas influenced the development of many modern neuropsychological batteries, primarily in the procedurally oriented development approach to psychometric testing [57]. This includes a two-step procedure which starts the testing with a standard kit (vertical section) with a subsequent limitation of the test procedures to the alleged defect in the second step (horizontal section). It is assumed that such a horizontal section will provide a fairly complete overview of the patient’s cognitive capacities.

Many neuropsychologists now use rather flexible approaches (not one standardized test battery, but separate tests with different batteries) to adequately fulfil the purpose of the neuropsychological examination and provide a patient with more integrative neuropsychological assessment. The standardization of the neuropsychological test battery began with Luria and the development of a protocol for neuropsychological assessments. It included elements of quantitative assessments of symptoms with a score of “no deficits, weak, moderate, or severe” [27]. Further research was aimed primarily at the differentiation of these criteria and the operationalization of the decision process. Currently, there are several options for standardization and quantification methods of Luria’s neuropsychological examination of both adults and children [58-62].

Specificity of the developed scoring system based on the qualitative analysis [59] lies in two related procedures which are distinct in value and applicability. First, results from each probe are compiled, and a list of possible disturbances is made. This makes it possible to identify a typical pattern and to qualify the parameters (symptoms) on the basis of their common relatedness to brain structures to compare the degree of deficit in different parts of the brain, and to determine the stability of symptoms during follow-up. Positive or negative alterations in the neuropsychological status during the second testing can be estimated on the basis of the disappearance/occurrence of disturbances. Scoring also makes it possible to determine the total score for each deficiency of each cognitive function and to estimate the dynamics of the subject’s status by longitudinal tracking during the rehabilitation procedure. Luria’s testing also allows reducing or expanding the testing procedure. The battery allows further improvement in formalization by applying procedures based on statistics, although in its present form it is without a doubt effective and useful for diagnostics also in neuropsychiatric facilities.

Thus, Luria’s style of evaluation comprises a single-case approach with a flexible set of testing procedures that allows a synthetic evaluation of the cognitive functions, making it possible to dissect cognitive function into several functional domains. Psychometric methods are focused on achievement, and provide an index score of the severity of cognitive impairment that facilitates the tracking of cognitive functioning during follow-up. When using evaluation of specific cognitive functions by standard psychometric tests two patients who have been evaluated with the same score severity may have qualitatively different cognitive disturbances. This might explain why experimental paradigms that frequently include multiple cognitive factors and performance on different tasks measuring cognitive processes often correlate weakly, reflecting the ambiguity of even well-known cognitive constructs [63]. Therefore, Luria’s approach, being focused on the process, allows the characterization of the types of mistakes and tracking of them throughout the testing procedure. The commonality of the deficits across the number of tasks may identify the impaired networks. Thus, Luria’s style of evaluation might prove especially helpful in tracking diffuse cognitive deficits caused by the disconnectivity of brain networks.

### Studies of cognition in schizophrenia using Luria’s test battery

Attempts to outline neuropsychological aspects of specific psychiatric disorders have grown substantially since mid1970’s [64,65]. The Luria-Nebraska Neuropsychological Battery (LNNB) has been one of the most widely used standardized and comprehensive tools in research on neuropsychological functioning. Neuropsychological research studies have long been concerned with the presence or absence of structural abnormalities in the brains of patients with schizophrenia, attempting to differentiate between their “organic” and “functional” deficits [66-69]. They were also performed together with common referral questions for psychiatric patients, which were used for differential diagnosis. Later on, there was an attempt to merge separate cognitive deficiencies into syndromes resulting in diverse cognitive profiles in different diagnostic categories and also speaking for the existence of associated brain mechanisms [70,71]. The studies often described the patients’ cognitive profiles, drawing attention to the particular disturbances within Luria’s functional blocks [72-76]. Their primary focus lay rather within the deficit specification than in the extent of disintegration of functional blocks and potential brain mechanisms.

### Luria revisited: an extension of neuropsychological performances

Along with the advancement of neuroimaging technologies, Luria’s systematic approach began to be applied as a conceptual framework. As an example, a method to study kinetic apraxia, initially proposed by Luria [27], the Fist-Edge-Palm-Test that requires sequential movements, is widely used in psychiatry, particularly for the study of neurocognitive deficits/neurological soft signs in schizophrenia. At the behavioural level, the disturbance in kinetic praxis may result in the inability to correct errors, which includes both the number of errors and the type of errors (errors of serial organization, such as individual mistakes, unstable tendencies of program expansion, inert expansion of the program, echopraxic movements and perseveration, and deautomatization). Motor programs require constant attentional control and can be linked to performance decrements found under dual-task conditions involving sensorimotor tasks. While a series of studies failed to demonstrate dual-task deficits involving two cognitive tasks, performance decrements in dual motor tasks have been described [77] which are indicative of attention-control decrements in schizophrenic patients under dual-task conditions and may be linked to prefrontal and motor pathways. Such phenomena have been observed in complex movements, specifically, rhythmic timing, and may be linked to elementary motor perseveration [78]. Interestingly, fMRI studies demonstrated activation of bilateral premotor and left parietal areas and the left cerebellum, as well as right sensorimotor and supplementary motor areas while performing the Fist-Edge-Palm Task [4,79]. As in a study of Rao et al., using psychophysiological interaction analysis (PPI), the authors found significant increases in functional connectivity between the bilateral sensorimotor cortex and the right inferior and middle frontal cortex during performance of the Fist-Edge-Palm Task in comparison to simple motor tasks (single palm tapping, pronation/supination task) [80]. These findings suggested a specific impairment in schizophrenia patients due to altered frontal connectivity, which is consistent with Luria’s original proposal. The traditional motor task developed by Luria has been incorporated into other tests. For example, the Neurological Evaluation Scale [81] and the Cambridge Neurological Inventory [82] have included several motor sequencing tasks designed by Luria for the evaluation of neurological signs in schizophrenia research. These signs are believed to target features [83] and endophenotypes [4,84] of schizophrenia. In particular, the Fist-Edge-Palm Task, has proven to be both sensitive and effective in discriminating patients with schizophrenia and individuals at risk for schizophrenia [85] as well as in discriminating of patients with schizophrenia and schizophrenia spectrum disorders from healthy controls [86].

Another application of Luria’s framework is the introduction of the taxonomy of executive functions based on neuropsychological perspectives and recent advancements in brain connectivity. The meta-analysis of Niendam et al. [87], refers to Luria’s ideas on the multidimensional structure of executive functioning that comprises the initiation of goal-directed activity, planning steps to achieve a goal, and subsequent controlling of a goal by inhibiting incorrect responses, as well as an additional domain of working memory that helps to maintain and manipulate information during task performance [88]. Lesion studies gave to the prefrontal cortex a pivotal role in executive functioning. However, neuroimaging studies have demonstrated that, depending on the task, other areas (posterior parietal areas and the anterior cingulate cortices) also become involved. A common pattern of activation of frontal-cingulate-parietal-subcortical connectivity supports the idea of the superordinate control network that is recruited during executive-task performance. Assuming that executive functioning is altered in schizophrenia, the proposed model and the contribution of the modular (prefrontal) versus shared (fronto-parietal connectivity) should be tested.

### Current views on cognition

Luria prefigured the direction of the cognitive neurobiological research. As he claimed, cognitive functions are dynamic systems characterized by nonlinearly interacting elements that organize into spatial and temporal patterns [27]. The interconnections between cognitive functions, like visual perception and attention [89], working memory and executive functioning [90] attention and working memory [91], and temporal processing [92] correspond to the present knowledge of brain connectivity. On a cellular level the interconnectivity of the brain indicates that there is closeness among different brain areas. The integrity of distinct neuronal structures is essential for the availability of specific cognitive functions [93]. Thus, one could expect that if one of the brain areas is functionally altered, it may affect other brain areas involved in the corresponding cognitive function.

Some of the diagnostic techniques, like electroencephalography (EEG), which depicts electrical activity in the brain, and magnetic resonance imaging (MRI), which measures the blood oxygen-level-dependent (BOLD) signal and serves as an indirect indicator of neuronal activity, are particularly sensitive to the delineation of brain areas. These techniques provide an insight into cognitive processes that are not readily studied by behavioral measures. The term “connectomics” was recently coined to define a set of neural connections forming the human brain [94]. Analyses of whole brain activity indicate that fairly all cognitive functions are associated with the activation of networks of widely distributed cortical areas rather than of individual specialized structure [95,96]. It has been postulated that cognitive operations emerge from coordinated activity in the distributed brain networks. Assuming the multidimensional organization of cognitive functions, the critical question that remains open is how to identify the relevant neural networks responsible for specific cognitive capacities, given that many brain regions are probably associated with a number of cognitive capacities. The one-to–one relation of brain regions to capacity, which is a core assumption of some proponents in cognitive neuropsychology [97], does not seem to apply to many psychiatric disorders, especially schizophrenia. Modern approaches based on brain-imaging research suggest rather complex structural-functional interactions.

Recent evidence suggests that functional connectivity is closely related to underlying structural connectivity [98]. Structural networks provide an anatomical framework for functional interactions [99]*.* In other words, we need to understand the structural connections in the brain to predict possible functional interactions. In areas that have a structural hierarchy, such as sensory networks, the functional activity pattern can be predicted. The interhemispheric coherence may be preserved by the active role of subcortical structures, including those of the brain stem [100].

Functional neuroimaging is being increasingly applied to the issues of connectivity and communication among different areas in the brain. In the absence of a task, the cerebral cortex generates rich and consistent spatio-temporal patterns of activity [101]. These spontaneously emerging fluctuations in the resting state appear to map the cortex and show amplitude similar to the fluctuations that are produced when performing a task [102]. One of the possible explanations is that the spontaneous fluctuations represent the readiness of the system to react to stimuli and keep the system close to the firing threshold [103].

A large body of neuroimaging studies have identified functional maps in various brain regions. Resting-state networks are slightly discrepant across different studies [104-107]. Although no classification has been explicitly proposed, M. Mesulam classifies networks according to their potential functional role in cognitive processing: a spatial attention network in the posterior parietal cortex and frontal eye fields, a language network in Wernicke’s and Broca’s areas, an explicit memory network in the hippocampal–entorhinal complex and inferior parietal cortex, a face-object recognition network in midtemporal and temporopolar cortices, and a working memory-executive function network in prefrontal and inferior parietal cortices [108]. Along with the specific, content-dependant networks, triple-theory networks were introduced. These large-scale networks include a salience network (SN) involving the dorsal-anterior cingulate and anterior insula regions, which mediate the selection of salient external and interceptive signals [109,110], a central executive network (CEN) consisting of regions in the middle and inferior prefrontal and parietal cortices engaged in many higher-level cognitive tasks and thought to be involved in adaptive cognitive control, and a default-mode network (DMN) consisting of regions in the medio-frontal cortex and posterior cingulate relating to resting-state or internally focused tasks that may be involved in attention to internal emotional states or self-referential processing [111]. The existing evidence supports a general role for the SN in switching between these CEN and DMN networks [110,112].

Another approach to identify multiple functional networks is to assign them to ‘processing’ or ‘control’ network categories [113], where processing-type networks are more static and modular, and control networks are dynamic and flexible and are able to adapt to various tasks. From the perspective of temporal dynamics, the high similarity in the relationships among brain areas within somato-motor and visual systems and the default-mode system might indicate that these systems are relatively stationary, whereas other networks, such as task-control systems, might have more dynamic connections [113]. Frontal-parietal networks (FPN) that include parts of the lateral prefrontal cortex and posterior parietal cortex are thought to be involved in modulation of the top-down control [114]. The role of these specific networks in different tasks that require control has not yet been clarified.

Current evidence suggests that brain networks may interact while performing certain cognitive tasks. Thus, positive correlations among functionally related brain regions and negative correlations among brain regions represent theoretically opposed functional roles of the networks. In particular, negative correlations have been observed between a set of regions routinely exhibiting increases in activity during attention-demanding tasks (task-positive regions) and a separate set of regions routinely exhibiting decreases in activity (task-negative regions) [115,116].

The notion that different functions are represented in different brain areas or have different algorithms which are interconnected leads to the question of how the activity of these different regions is temporarily coordinated. It has also been suggested that cognitive processing occurs through a stream of discrete units or epochs rather than as a continuous flow of neuronal activity, i.e. that mental activity evolves through a sequence of quasi-stable coordination states [29,117,118]. EEG and direct neuronal recording [119,120], have documented that cortical neurons belonging to specific, but spatially separated, functional clusters show correlated patterns of spontaneous activity over time during resting states. The topographic representation of the EEG scalp electrical field affords temporal resolution at the millisecond level, yet does not change randomly or continuously over time, remaining stable over periods of approximately 100 ms. Such quasi-stable and unique topographic distributions of the electrical-field potential have been termed “microstates” [121,122]. Accordingly, parsing the EEG data stream into microstates should lead to a putative dictionary of functional brain units. Different types of cognition have been found to be associated with different types of microstates [123] and suggest microstates as potential candidates for basic psychophysiological units of cognition.

In a taxonomy of functions proposed by E. Pöppel which is based on neuropsychological observations, a distinction is made between “what-functions” representing the content of consciousness, and “how-functions” representing the logistics of neuronal processing (like temporal control, attention or activation) [124]. Both functional domains are necessary and sufficient for cognition. Their temporal coordination is provided by neuronal coordination mechanisms, which are expressed as oscillatory processes in neuronal populations. On the basis of such neuronal oscillations, the brain can provide itself with independent temporal states. Temporal processing in the brain is hierarchically organized, and different mechanisms are responsible for the transitions from simultaneity to non-simultaneity, to temporal order, to the subjective present, and to the estimation of duration. Each next step requires successful processing at the lower processing levels [29,93]. The Russian physiologist I.M. Sechenov emphasized the role of time in auditory and proprioceptive sensory systems (in contrast to the perception of space, where the leading role is given to vision and skin sensitivity) [125]. For many years, the spatial connections and the temporal coordination of cognitive functions were difficult to grasp, but neuroimaging methods present new possibilities to study the precise mechanisms of cognitive processing.

Assuming a two-dimensional model of cognitive functions, as well as their hierarchical organization, cognition must be examined from the perspective of spatio-temporal connectivity. Large-scale networks might correspond to the proposed “neuronal workspace” that consists of a distributed set of cortical neurons that form a discrete spatio-temporal pattern of activity [126,127]. Functional networks may be organized according to a hierarchy of temporal scales driven by structural and functional connections and supporting the existence of a hierarchical functional organization across time scales [128].

## Conclusion

### Future directions

The development of current neuropsychology reflects the universal trend to substitute the static approach focused on specific brain lesions for a dynamic approach that embodies the dynamics of brain-behavior interactions. In psychiatry, this issue is becoming central, as many psychiatric disorders present with subtle structural and robust functional brain alterations. Luria’s approach, which was initially introduced in neurology, made a substantial contribution not only to the diagnostics of cognitive symptoms and the development of neurorehabilitation strategies, but also pioneered a theoretical concept of cognition as a dynamic system. Due to the growing necessity for operationalized cognitive criteria in psychiatry, the major role in the evaluation of cognitive functions has been given to psychometric tools, which provide a comprehensive measurement of the severity of cognitive dysfunction. Nowadays, psychometric tests are often preferred to Luria’s neuropsychological evaluation, that is oriented to the process and provide only limited scoring for the evaluation. The clinical test utility as well as the conceptual value of Luria’s systematic approach was overlooked. In the scope of the theoretical and practical issues in psychiatry, Luria’s concept of cognitive functions as dynamic systems should be revisited.

From the theoretical standpoint, the major benefit of Luria’s systematic approach lies in well-structured and well-defined integrative cognitive components and contains information about their possible interactions. It provides a theoretical framework for the development of a new taxonomy of cognitive functions desperately needed in psychiatry and cognitive neuroscience.

Patients with schizophrenia tend to have a less strongly integrated, more diverse profile of functional brain connectivity. Alongside the cognitive deficits, however, brain networks in schizophrenia patients are robustly repeated, pointing to a possible benefit of the schizophrenia connectome [129]. It is absolutely critical in the field of psychopathology to examine interactions between systems, as the complexity and range of impairments present in schizophrenia are hardly due to impairments in a single system [130]. In this regard, Luria’s neuropsychological measures can help in making inferences about the cognitive processes related to certain brain networks.

Therefore, Luria’s conceptual framework of interconnections and its application in evaluative measures represent potentially constructive contributions to current psychiatric research in schizophrenia. From the practical standpoint, the qualitative evaluation of the cognitive domains might be beneficial for differential diagnosis and prognosis.

In Luria’s own opinion, his most important accomplishment was the theory of complex dynamic systems in neuropsychology [53]. Since the investigation of specific disturbances of cognitive functions is very important for understanding the aetiology and pathogenesis of schizophrenia, it is essential to take into account the results of their experimental evaluation. The ingenuity of Luria’s qualitative analyses may help to do so and continue to influence future neuropsychological studies in schizophrenia and psychiatry in general.

### Endnote

^a^Alexander Romanovich Luria (1902–1977) is universally recognized as one of the distinguished psychologists of our time. Born on July 16, 1902 in the city of Kazan, Russia, Luria first studied at the local university, and was influenced by the works of Wundt, Ebbinghaus, and Freud. In 1922, he was invited to the Moscow State University, where he joined Alexey N. Leontiev’s group and met Lev Vygotsky, whose influence on the development of a systematic theory of neuropsychology he considered to be “large and essential”. From his studies of localized brain lesions at the Rehabilitation Hospital of Nervous Diseases in Kisegach in the Southern Urals during World War II and later at the Moscow Institute of Neurosurgery, Luria initiated the field of neuropsychology. His international acclaim was confirmed in a series of publications and at conferences in the post-Stalinist era. In 1973 he became a member of the National Academy of Sciences of the United States. Luria continued his work at the Moscow Institute of Neurosurgery and the Moscow State University and remained highly productive into old age. He died in Moscow on August 14, 1977. The introduction of higher cortical functions as compound dynamic systems was made by A.R. Luria (1973) [25], who established the system-dynamic approach based on empirical studies and clinical observations (lesion studies).

## Abbreviations

BOLD Level: Blood Oxygen-Level-Dependent Level
CAS: Cognitive Assessment System
CEN: Central Executive Network
DMN: Default Mode Network
DSM-5: Diagnostic and Statistical Manual of Mental Disorders, Fifth Edition
EEG: Electroencephalography
FPN: Fronto-Parietal Network
LNNB: Luria-Nebraska Neuropsychological Battery
fMRI: Functional Magnetic Resonance Imaging
MRI: Magnetic Resonance Imaging
PASS: Planning, Attention, Simultaneous, Successive
PPI: Psychophysiological Interactions Analysis
SN: Salience Network
RSNs: Resting State Networks
VBM: Voxel-Based Morphometry
